# Evaluation of the Adenocarcinoma-Associated Gene *AGR2* and the Intestinal Stem Cell Marker *LGR5* as Biomarkers in Colorectal Cancer

**DOI:** 10.3390/ijms13044367

**Published:** 2012-04-05

**Authors:** Manuel Valladares-Ayerbes, Moisés Blanco-Calvo, Margarita Reboredo, María J. Lorenzo-Patiño, Pilar Iglesias-Díaz, Mar Haz, Silvia Díaz-Prado, Vanessa Medina, Isabel Santamarina, Sonia Pértega, Angélica Figueroa, Luis M. Antón-Aparicio

**Affiliations:** 1 Medical Oncology Department, La Coruña University Hospital, Servicio Galego de Saúde (SERGAS), As Xubias, 84. PC 15006, La Coruña, Spain; E-Mails: margarita.reboredo.lopez@sergas.es (M.R.); luis.miguel.anton.aparicio@sergas.es (L.M.A.-A.); 2 Translational Cancer Research Lab, Biomedical Research Institute (INIBIC), Carretera del Pasaje, s/n. PC 15006, La Coruña, Spain; E-Mails: moises.blanco.calvo@sergas.es (M.B.-C.); maria.del.mar.haz.conde@sergas.es (M.H.); vanessa.medina.villaamil@sergas.es (V.M.); isabel.santamarina.cainzos@sergas.es (I.S.); angelica.figueroa.conde-valvis@sergas.es (A.F.); 3 Pathology Department, La Coruña University Hospital, Servicio Galego de Saúde (SERGAS), As Xubias, 84. PC 15006, La Coruña, Spain; E-Mails: maria.lorenzo.patino@sergas.es (M.J.L.-P.); pilar.iglesias.diaz@sergas.es (P.I.-D.); 4 Tissue Engineering and Cellular Therapy Lab, INIBIC, Carretera del Pasaje, s/n. PC 15006, La Coruña, Spain; E-Mail: silvia.ma.diaz.prado@sergas.es; 5 Medicine Department, La Coruña University (UDC), Campus de Oza, s/n, PC 15006, La Coruña, Spain; 6 Biostatistics and Clinical Epidemiology Unit, La Coruña University Hospital, Servicio Galego de Saúde (SERGAS), As Xubias 84, PC 15006, La Coruña, Spain; E-Mail: sonia.pertega.diaz@sergas.es

**Keywords:** colorectal cancer, real-time PCR, circulating tumor cells, prognostic markers, stem cells, anterior gradient homolog-2, leucine-rich repeat-containing G-protein-coupled receptor 5

## Abstract

We aim to estimate the diagnostic performances of anterior gradient homolog-2 (*AGR2*) and Leucine-rich repeat-containing-G-protein-coupled receptor 5 (*LGR5*) in peripheral blood (PB) as mRNA biomarkers in colorectal cancer (CRC) and to explore their prognostic significance. Real-time PCR was used to analyze *AGR2* and *LGR5* in 54 stages I-IV CRC patients and 19 controls. Both mRNAs were significantly increased in PB from CRC patients compared to controls. The area under the receiver-operating characteristic curves were 0.722 (*p* = 0.006), 0.376 (*p* = 0.123) and 0.767 (*p* = 0.001) for *AGR2*, *LGR5* and combined *AGR2/LGR5*, respectively. The *AGR2/LGR5* assay resulted in 67.4% sensitivity and 94.7% specificity. *AGR2* correlated with pT3–pT4 and high-grade tumors. *LGR5* correlated with metastasis, R2 resections and high-grade. The progression-free survival (PFS) of patients with high *AGR2* was reduced (*p* = 0.037; HR, 2.32), also in the stage I-III subgroup (*p* = 0.046). *LGR5* indicated a poor prognosis regarding both PFS (*p* = 0.007; HR, 1.013) and overall survival (*p* = 0.045; HR, 1.01). High *AGR2/LGR5* was associated with poor PFS (*p* = 0.014; HR, 2.8) by multivariate analysis. Our findings indicate that the assessment of *AGR2* and *LGR5* in PB might reflect the presence of circulating tumor cells (CTC) and stem cell like CTC in CRC. Increased *AGR2* and *LGR5* are associated with poor outcomes.

## 1. Introduction

Colorectal cancer (CRC) is one of the leading causes of cancer-associated morbidity and mortality across the world. The predicted number of deaths in 2011 in the European Union due to CRC was 162,026 [1]. The stage at diagnosis and the possibilities for curative surgery remain the most important prognostic factors.

The development of blood-borne metastasis is ultimately responsible for most CRC-related deaths. Sensitive methods to detect circulating tumor cells (CTC) could serve as prognostic or predictive tools to identify patients at a high risk of disease progression who could be selected for additional treatment [2].

CTC are identified mainly by using antibodies against epithelial antigens or molecular approaches. The PCR amplification of tissue- or tumor-specific mRNA is commonly used to detect circulating or occult metastatic cells. Systematic reviews, meta-analyses and prospective studies [3–7] provide coherent evidence that the molecular detection of CTC in the peripheral blood (PB) is of strong prognostic significance in patients with CRC.

Our study aimed to evaluate promising CRC-specific mRNAs for multi marker detection of CTC in PB. We previously [8,9] identified anterior gradient homolog-2 (*AGR2*) and plakophilin-3 as potential CTC markers in gastrointestinal cancer through an in silico profile of gene expression and quantitative real-time reverse-transcription PCR (qRT-PCR). Moreover, *AGR2* has been included in the molecular signature that defined CTC in metastatic breast, prostate and colorectal cancers [10,11].

*AGR2* encodes a 17 kDa secreted protein, homologue of the Xenopus cement gland gene *XAG-2* [12]. Although its functions in humans are poorly understood, recent reports indicate that AGR2 can induce cellular transformation and tumor growth, promote cell survival through inhibition of p53, enhance tumor cell adhesion to the substratum and enhance cell migration [13–15].

Recent data [2,10,11,16,17] suggest that CTC encompass a heterogeneous cell population with different tumorigenic capabilities and include cells characterized by an epithelial-mesenchymal plasticity (EMP) with transient loss of epithelial markers. In that sense, the use of different mRNA biomarkers will yield better results in the identification of CTC and rare cell subsets of biological relevance. Thus, it has been hypothesized that only CTC with tumor-initiating properties will eventually complete the metastatic cascade and will develop clinically relevant metastases [18].

The leucine-rich repeat-containing G-protein-coupled receptor 5 (*LGR5*) also known as G-protein-coupled receptor 49 (*GPR49*), has been recently reported as a marker for stem cells (SC) in the small intestine and colon [19]. Recently [20,21] it was shown that the *LGR5* gene and protein were markedly over expressed in the majority of advanced CRCs and in CRC cell lines derived from metastatic tumors. Moreover, high *LGR5* expression has been associated with poor progression-free survival for CRC patients [22].

Thus, we hypothesized that *LGR5* mRNA expression in PB of CRC patients could indicate the presence of circulating tumor cells with stem cell properties.

The primary aims of our study were to estimate prospectively the diagnostic accuracy and usefulness of *AGR2* mRNA in PB as a surrogate biomarker of CTC and to explore its prognostic significance. Additionally, the blood expression of the intestinal stem-cell (ISC) marker *LGR5* was evaluated for correlations with *AGR2* and clinical parameters. Our findings revealed that molecular assessment of *AGR2* and *LGR5* can serve as a marker of CTC and ISC-like CTC in CRC patients, which underscores their potential clinical relevance as predictors of disease outcome.

## 2. Results and Discussion

### 2.1. Results

#### 2.1.1. Patients and Clinical Data

Starting in July 2004, 54 patients with histological proven CRC and 19 controls were consecutively recruited for this study. This sample size allowed us to estimate an expected area under the ROC curve of 0.70 with a standard error of 0.065. Ninety per cent of the subjects were included within the first two years. The clinical characteristics of the patients are shown in Table 1.

The mean age was 62.2 years (SEM 1.84; median, 62 years; range, 43 to 74 years) in the control group and 62.7 (SEM 1.30; median, 62.5; range, 31 to 80 years) in the patient group (*t* test, *p* = 0.847). The ratio of males to females was similar in the controls (men 63.2%) and the patients (men 61%) (χ^2^ test, *p* = 0.875).

PB samples were obtained after R0 or R1 surgery in 16 patients. In 38 patients, blood samples were obtained before neo-adjuvant chemotherapy or in the presence of active metastatic disease, both of which were categorized as R2. In patients with node-negative disease and R0 resection, the mean number of lymph nodes analyzed was 12.8 (SEM 2.7; range 7–21).

Patients with metastatic CRC (*n* = 38) were grouped into high- (19.4%), intermediate- (36.1%) and low-risk groups (44.4%) using performance status, number of tumor sites, alkaline phosphatase and white blood cell count, as suggested by Köhne *et al*. [23] Median overall survival (OS) and progression-free survival (PFS) were 98 and 39 weeks, 56 and 26 weeks, and 59 and 14 weeks for the low-, intermediate- and high-risk groups, respectively. The median OS tended to be higher (log-rank *p* = 0.061) in the low-risk group (98 weeks; 95% CI, 43.1 to 152.9) compared to the combined intermediate/high-risk group (56 weeks; 95% CI, 47.2 to 64.8).

All patients were followed up until death or the end of the study. Disease progression events occurred in 39 patients (72.2%). There were three relapses among stage I–III patients and 36 progressions of metastatic disease. The median PFS was 44 weeks (95% CI, 24.8 to 63.2 weeks). The median OS was 132 weeks (95% CI, 84.4–179.6 weeks), and 34 patients (63%) died of advanced disease. The mean (SEM) follow-up time for the patients still alive at the time of the analysis was 232 (17.8) weeks (median, 232.5 weeks; range, 67 to 335 weeks).

#### 2.1.2. Expression of *AGR2* and *LGR5* mRNA Transcripts in Blood Samples

*AGR2* mRNA was quantified in 62 blood samples (84.9%), including 43 samples obtained from patients with CRC and 19 from controls. The *LGR5* mRNA level was quantified in 67 blood samples (90.5%), 48 from CRC patients and 19 from controls. mRNA was insufficient or its quality was inadequate for qRT-PCR in 11 (15.1%) and 6 (8.2%) patients’ samples for *AGR2* and *LGR5* respectively.

The mean relative *AGR2* mRNA expression was 29.1 (SEM 28.2; median 0.77; range, 0.21 to 536.7) in controls and 418.57 (SEM 84.4; median 191.2; range, 0.05 to 1989.5) in cancer patients (*t* test, *p* < 0.001). Likewise, the *AGR2* level was significantly increased (ANOVA, *p* = 0.007) in patients with stage IV CRC (mean 492.6; SEM 114) compared with stage I to III patients (mean 305.4; SEM 122.5) and non-cancer controls (mean 29.1; SEM 28.2).

The mean *LGR5* mRNA level was 0.21 (SEM 0.03; median 0.18; range, 0 to 0.4) in controls and 11.6 (SEM 4.9; median 0.08; range, 0.01 to 146.9) in patients (*t* test, *p* = 0.026). The *LGR5* level was significantly increased (ANOVA, *p* = 0.038) in patients with stage IV CRC (mean 18.40; SEM 7.70) compared with stage I to III patients (mean 0.20; SEM 0.06) and non-cancer controls (mean 0.21; SEM 0.03). There was no correlation between *AGR2* and *LGR5* blood levels in the patients group (Pearson correlation coefficient −0.009; *p* = 0.952).

**Figure 1:**
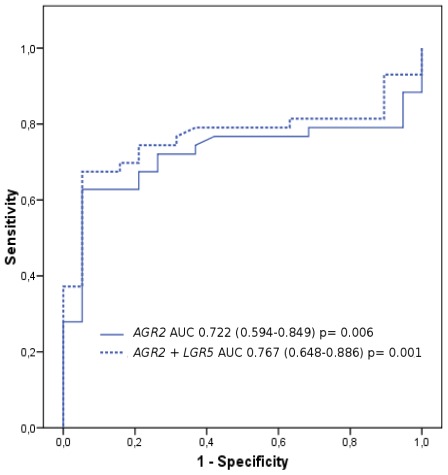
*AGR2* and *LGR5* ROC curves. mRNA relative levels were quantified in blood obtained from patients with colorectal cancer and from controls. Area under the curve (AUC), 95% confidence interval and *p*-values are shown.

ROC curves of circulating mRNAs were constructed in order to be able to discriminate different groups (Figure 1).

Comparing the relative *AGR2* levels in patients and controls, the AUC was 0.722 (95% CI, 0.594–0.849; *p* = 0.006). According to the ROC curve, a relative level for *AGR2* mRNA in the blood of 1.65 was defined as the optimal cutoff value (Youden index) for differentiating patients with CRC from the controls. With this cutoff value for *AGR2*, the sensitivity and specificity of 62.8% (95% CI, 46.7 to 76.6) and 94.7% (95% CI, 71.9 to 99.7) respectively, were achieved. At this threshold value, *AGR2* positivity was associated with CRC diagnostic (*p* < 0.001).

The ROC curve for *LGR5* showed an AUC of 0.376 (95% CI, 0.233–0.520; *p* = 0.123). A relative blood level of 0.39 was defined as the optimal cutoff point for *LGR5*. With this cutoff value, the sensitivity and specificity for the *LGR5* mRNA assay were 18.8% (95% CI, 9.4 to 33.10) and 100% (95% CI, 79.1 to 99.5) respectively. At this cutoff value, *LGR5* positivity tended to associate with CRC diagnostic (*p* = 0.052).

In CRC patients, relative expression values for *AGR2* and/or *LGR5* in blood above these cutoff points, defined as the Youden index, were found in 16.7% of stage I–II, in 72.7% of stage III and in 76.9% of stage IV patients (χ^2^ test; *p* = 0.016).

*AGR2* and *LGR5* markers were analyzed in combination by logistic regression. The predicted probabilities of diagnosis generated a ‘combination marker’ ROC curve. The combination (*AGR2*/*LGR5*) had an AUC-ROC = 0.767 (95% CI, 0.648–0.886; *p* = 0.001) which was slightly improved [24] compared to *AGR2* alone (*p* = 0.25). The sensitivity and specificity of the combination were 67.4% (95% CI, 51.3 to 80.5) and 94.7% (95% CI, 71.9 to 99.7) respectively (Figure 1).

#### 2.1.3. Clinic Pathological Characteristics and mRNA Markers in Blood

The clinical and pathological characteristics and the *AGR2* and *LGR5* mRNA expression in blood from cancer patients are shown in Table 2.

A significant higher relative level of *AGR2* blood expression was found in pT3-T4 tumors (*p* = 0.002) and high-grade lesions (*p* = 0.023). There was a tendency (*p* = 0.063) to higher *AGR2* levels associated with lymph node metastasis. Increased *LGR5* expression was found in patients (Table 2) with stage IV (*p* = 0.024), R2 resections (*p* = 0.024) or high-grade tumors (*p* = 0.024).

Carcinoembryonic antigen (CEA) and carbohydrate antigen 19.9 (CA 19.9) serum levels were increased above the upper limits of normal in 46.3% and 38.9% of the patients, respectively. There were no correlations between *AGR2* or *LGR5* mRNA levels with CEA or CA 19.9 in serum (Pearson −0.172, −0.155, 0.021 and −0.063 respectively).

To explore the possible influence of recent surgery on the circulation of tumor cells, we analyzed *AGR2* and *LGR5* levels according to the time interval from operation and blood sampling. The mean time from surgery to blood sampling for mRNA quantification was 52.5 weeks (SEM 8.7 weeks; median, 18 weeks; range, 1 to 202 weeks). The 25th percentile was 6.75 weeks. There was no significant difference in *AGR2* and *LGR5* levels between time intervals (<6.75 or ≥6.75 weeks) from the last surgery.

In the group of patients with stage IV disease, *AGR2* and *LGR5* were analyzed according to the prognostic subgroups defined as described previously [19]. The mean (SEM) relative *AGR2* levels were 443.1 (229.6) and 518.8 (129.9) for low- and combined intermediate/high-risk groups, respectively (*t* test, *p* = 0.759). The median (SEM) relative *LGR5* levels were 15.5 (11.7) and 20.6 (10.4) for low- and combined intermediate/high-risk groups, respectively (*t* test, *p* = 0.746).

#### 2.1.4. Prognostic Significance of *AGR2* and *LGR5* in Blood

To analyze the relationships between biomarker expression and outcomes (PFS and OS) we estimated the hazard ratios associated with mRNA levels as continuous variables using Cox regression models [25]. There was a trend for a high risk of disease progression associated with increased *AGR2* relative blood expression (HR 1.0; 95% CI, 1.0 to 1.001; *p* = 0.093). There was no association with the risk of death (HR 1.0; 95% CI, 0.999 to 1.001; *p* = 0.913). However, in stage I to III patients, the risk of disease progression was higher with increasing *AGR2* level (HR 1.002; 95% CI, 1 to 1.004; *p* = 0.046).

Increasing relative blood expression of *LGR5* mRNA as a continuous variable was associated with a higher risk of disease progression (HR 1.013; 95% CI, 1.004 to 1.023; *p* = 0.007). The risk of death was also higher with increasing levels for *LGR5* mRNA in the blood (HR 1.01; 95% CI, 1 to 1.020; *p* = 0.045).

**Figure 2:**
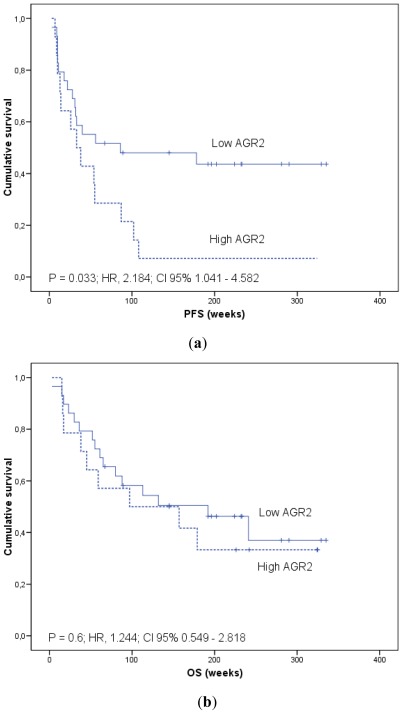
*AGR2* and survival analysis. Kaplan-Meier plots of (**a**) progression-free survival (PFS) and (**b**) overall survival (OS) in colorectal cancer patients according to *AGR2* mRNA expression in blood. Relative quantification of *AGR2* mRNA was calculated by the 2^−ΔΔCt^ method using HPRT as a reference gene. Continuous mRNA levels were converted to a dichotomous variable using the mean levels of expression as a threshold. p estimates by log-rank test. Hazard ratios (HR) were modeled using Cox proportional hazard regression analysis.

**Figure 3:**
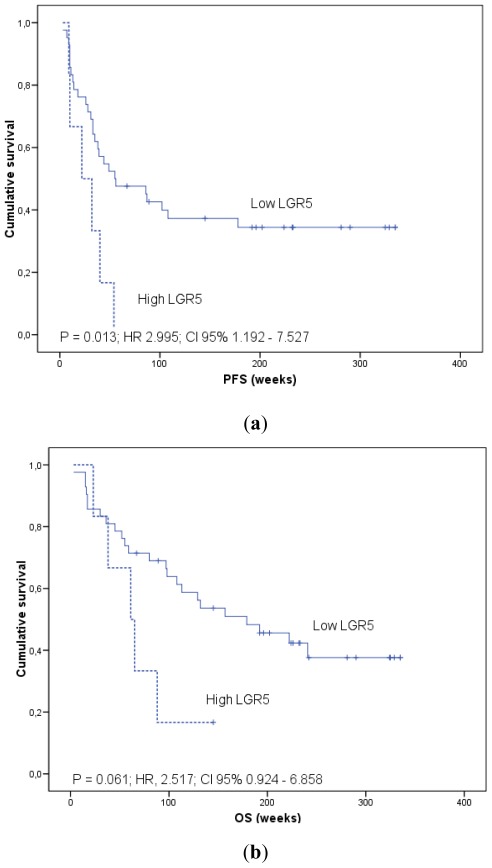
*LGR5* and survival analysis. Kaplan-Meier plots of (**a**) progression-free survival (PFS) and (**b**) overall survival (OS) in colorectal cancer patients according to *LGR5* mRNA expression in blood. Relative quantification of *LGR5* mRNA was calculated by the 2^−ΔΔCt^ method using HPRT as a reference gene. Continuous mRNA levels were converted to a dichotomous variable using the mean levels of expression as a threshold. p estimates by log-rank test. Hazard ratios (HR) were modeled using Cox proportional hazard regression analysis.

**Figure 4:**
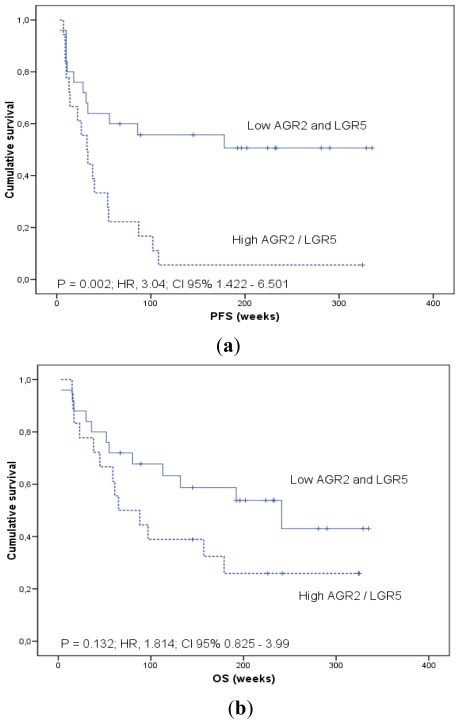
Combined *AGR2/LGR5* and survival analysis. Kaplan-Meier plots of (**a**) progression-free survival (PFS) and (**b**) overall survival (OS) in colorectal cancer patients according to combined *AGR2/LGR5* mRNA profile in blood. Relative quantification of mRNA was calculated by the 2^−ΔΔCt^ method using HPRT as a reference gene. Patients were divided into favorable mRNA profile (both *AGR2* and *LGR5* below the mean) and unfavorable mRNA profile (*AGR2* and/or *LGR5* above the mean). p estimates by log-rank test. Hazard ratios (HR) were modeled using Cox proportional hazard regression analysis.

In addition, in order to generate survival curves, we converted continuous mRNAs expression levels measured on qRT-PCR to a dichotomous variable, using the mean levels of expression in the patients group as a threshold. Kaplan-Meier curves for patients categorized according to *AGR2* and *LGR5* mRNA expression in blood are shown (Figures 2–4).

The median PFS for the group with high *AGR2* blood expression were 33 weeks (95% CI, 11 to 55) compared with 86 weeks (95% CI, 0 to 305.1) in the group with low *AGR2* (log-rank test, *p* = 0.033). Patients with high *AGR2* showed worse OS (median 97 weeks; 95% CI, 0 to 262.9) compared with those with low *AGR2* expression (median 192 weeks; 95% CI, 56.6 to 327.4) although this difference was not statistically significant (log-rank test, *p* = 0.6) (Figure 2).

Analysis of the patients’ outcome according to *LGR5* blood expression revealed that the high *LGR5* group exhibited significantly worse PFS (median 22 weeks; 95% CI, 0 to 48.4) compared with patients in the low *LGR5* group (median 55 weeks; 95% CI, 5.1 to 104.9) (*p* = 0.013). Although non-significant, there was a trend (*p* = 0.061) for a better OS in the group of patients with low *LGR5* (median 179 weeks; 95% CI, 74.9 to 283.1) compared with the group with increased *LGR5* blood levels (median 61 weeks; 95% CI, 28.6 to 93.4) (Figure 3).

High mRNA in PB (combined *AGR2* and/or *LGR5* transcript above the threshold cutoff) was found in 0, 36.4% and 53.8% of stage I–II, III and IV patients, respectively (χ^2^ test; *p* = 0.05).

Patients were divided into favorable mRNA profile (both *AGR2* and *LGR5* below the mean) and unfavorable mRNA profile (*AGR2* and/or *LGR5* above the mean). At the time of analysis, the mean and the median PFS in the favorable group were 190.8 weeks (95% CI, 131.2 to 250.4 weeks) and not reached in the unfavorable group. The mean and the median PFS were 54.7 weeks (95% CI, 21.2 to 88.1 weeks) and 32 weeks (95% CI, 17.5 to 46.6 weeks) in the unfavorable mRNA profile group (log-rank test *p* = 0.002) (Figure 4).

Multivariate Cox regression analyses were performed to determine whether high mRNA in blood were independently statistically predictive of PFS or OS (Table 3).

In testing for the independent prognostic significance of high *AGR2*/*LGR5* expression in a model with pT depth of invasion, lymph node involvement and residual disease (R resection status), the R status (HR of recurrence, 5.8; 95% CI, 1.7 to 19.7; *p* = 0.005) and the high mRNA blood expression (HR, 2.8; 95% CI, 1.2 to 6.4; *p* = 0.014) remained associated with PFS (Table 3). In this model, the only factor that retained independent prognostic significance for OS was R2-residual disease (HR of death, 7.338; 95% CI, 1.683 to 31.985; *p* = 0.008).

### 2.2. Discussion

Highly sensitive detection of CTC and detailed molecular characterization of rare cancer cell subpopulations may not only provide insights into the biology of early metastatic spreading, but these tools can also potentially indicate substantial predictive or prognostic information. PCR amplification of tumor mRNA is a powerful analytical tool for surrogate detection and characterization of CTC. Real-time RT-PCR allows for quantification of the tumor cell load in the PB and, at least theoretically, the determination of cutoff values of mRNA expression of clinical relevance in cancer patients. However, the sensitivity and specificity of this approach both depend on the expression level of candidate biomarkers in tumor cells as well as their background expression in the blood [26,27].

Evidence is rapidly accumulating that cancers are composed of heterogeneous populations of cells. Thus, one would predict that CTC might be enriched in cancer cells that express those biomarkers indicating the greatest invasive and metastatic capacity, including cancer stem cells (CSC) markers. Hence, the selection of appropriate target mRNAs that may be useful for clinical detection of CTC and CSC remains an important outstanding issue.

The current study was intended to assess the diagnostic performance of quantitative RT-PCR detection of *AGR2* in the blood as a surrogate marker of CTC. We then hypothesized that a marker indicative of the phenotype of colonic stem cells, such as *LGR5*, would improve the detection of biologically and clinically relevant CTC.

We found that *AGR2* mRNA was significantly elevated in the blood of patients with CRC compared to controls. ROC analysis suggested that at 94.7% specificity, *AGR2* achieved 62.8% sensitivity in distinguishing CRC blood samples from the control group. Furthermore, in CRC patients, blood *AGR2* mRNA levels correlated with different pathological prognostic factors, including pT3–pT4 depth of invasion and high-grade tumors.

These results are in line with the current evidence indicating that *AGR2* can promote cancer growth, cell survival, migration and anchorage-independent growth and cellular transformation [14,28]. In the clinical setting, *AGR2* protein expression in the primary tumor is an independent prognostic indicator of poor outcome in patients with breast [29] and prostate adenocarcinomas [30], and one recent study showed that increased *AGR2* protein in plasma is associated with ovarian cancer [31].

However, to the best of our knowledge, no comprehensive report has been published about the potential prognostic relevance of *AGR2* in colorectal cancer. Our findings indicate for the first time that the quantitative assessment of *AGR2* mRNA in blood might indicate a poor patient outcome in CRC. Remarkably, in stage I to III patients, the risk of disease progression was higher with increasing levels of *AGR2* in the blood. Likewise, in CRC patients with high *AGR2* blood expression, the PFS was significantly reduced, and there was a numerical but non-significant inferior OS.

A recent study [32] demonstrates that *AGR2* induces the expression of the growth-promoting EGFR ligand amphiregulin in human adenocarcinomas. This effect is mechanistically mediated through Yes-associated protein (YAP1) dephosphorylation. Interestingly, YAP1 is also implicated in the regulation of stem cell division through the repression of the Hippo pathway. These data and a previous report [14] show that proliferating and non-proliferating ISCs, as well as transit-amplifying cells from a secretory lineage express *AGR2* and suggest additional mechanisms for oncogenic actions for *AGR2*.

We next explored the expression of the ISC marker *LGR5* in the blood of our cohort of controls and CRC patients. We found that *LGR5* mRNA was significantly elevated in the blood of patients with colorectal carcinoma compared to controls. However, mean levels of *LGR5* mRNA were similar in controls and early stage CRC patients. Nevertheless, there was a significant increase of *LGR5* in blood obtained from metastatic CRC patients. When a cutoff point was defined based on the ROC curve, the *LGR5* assay achieved only 18.8% sensitivity but 100% specificity in distinguishing CRC and control blood samples. Conversely, *LGR5* mRNA in the blood showed a significant correlation with high-grade tumors, metastatic disease and R2 resections. Likewise, *LGR5* expression in the blood showed a prognostic value regarding both PFS and OS in CRC patients, as suggested by the Cox regression and Kaplan-Meier analysis. In that sense, our results suggested that *LGR5* is expressed only in a rare subset of CTC possibly including cancer stem-like cells. We could speculate that these circulating *LGR5*-expressing cells might contribute to cancer progression and therapeutic response.

The clinical and biological significances of *LGR5* expressing-cells in CRC are poorly understood. A primary tumor profile that encompasses known ISC markers, such as *LGR5*, has been strongly associated both with CRC stages and the occurrence of tumor relapse and metastasis [33]. *LGR5* protein expression had been associated with a poor PFS in CRC patients [22]. In contrast, in a recent report [34] a gene signature defined by methylation silencing of the Wnt-driven ISC marker genes, including *LGR5*, in CRC tumors was associated with a poor prognosis.

A number of proposed CSC markers, such as CD44 and CD133, have been explored in CTC detection. Recently, Iinuma H. *et al*. [7] demonstrated in patients with Dukes’ stage B and C CRC that the detection of CEA/Cytokeratins (CK) 19/20/CD133 mRNA in blood was useful for determining which patients were at high risk for recurrence and poor prognosis. However, in the CD133 single-marker analysis, no significant differences in OS and PFS were found [7]. In metastatic CRC, the transcriptional amount of CD133 in blood before resection of hepatic metastases resulted in a high risk of dying of recurrence after apparently curative liver surgery [35]. Nonetheless, CD133 and other putative markers for CRC stem cells such as CD44 are also expressed in a variety of cells including hematopoietic and/or endothelial cells (reviewed Hundt, S. in [27]), a factor that could diminish their specificity. The expression patterns of LRG5 and colon differentiation markers such as cytokeratin−20 are mutually exclusive [33] are of special interest for CTC detection. These facts strengthen the relevance of non-CK mRNA biomarkers for the detection of the most aggressive and specific subpopulations of CTC in CRC patients.

CTC in gastrointestinal cancer patients are increasingly detected when blood is obtained per- or intra-operatively [36]. However, the postoperative sampling time might reflect the most relevant CTC status [4,37]. In our study, blood samples were obtained several weeks after surgery. In order to explore the possible influence of recent surgery on the circulation of tumor cells, *AGR2* and *LGR5* levels were analyzed according to time intervals between surgery and blood sampling; conversely, no significant differences in biomarker mRNA levels between time intervals were found. From a clinical perspective, assessment of baseline prognostic factors and CTC detection rates may be of interest. In previous studies [38,39] including patients with metastatic CRC, the number of CTC detected using the Cell Search System was associated with high LDH level, liver metastasis and poorer performance status. Hence, we performed an exploratory analysis in the subset of stage IV CRC patients, which showed no association between a positive mRNA result and baseline clinical prognostic subgroups categorized according to performance status, white blood cell count, alkaline phosphatase and number of metastatic sites. In addition, levels of *AGR2* and *LGR5* were not significantly different either.

The combined *AGR2* and *LGR5* assay resulted in an increased sensitivity (67.4%; AUC-ROC = 0.767; *p* = 0.001) to separate cancer patients and controls. Remarkably, and in spite of the limited number of patients, Cox multivariate analysis demonstrated that *AGR2*/*LGR5* mRNA detection was a significant prognostic factor for PFS (HR, 2.8; 95% CI, 1.2 to 6.4; *p* = 0.014). Thus, the transcriptional amount of *AGR2*/*LGR5* in the PB defined subgroups of CRC patients with significantly different risks of disease progression, improving the so-called biologic specificity [40] of CTC detection.

Our findings indicate a high sensitivity and specificity for *AGR2*/*LGR5* qRT-PCR for the surrogate detection of CTC in PB samples and it could be useful as a prognostic factor in patients with CRC. However, taking into account the design and sample size of the study, the outcome results could only be considered as generating a hypothesis. Additional possible limitations of this study must be considered. Although the inclusion of patients with different stages and residual tumor status could be considered limitations of the study, we suggest that this pragmatic design accurately reflects the patients attending the oncology clinic every day. Thus, the diagnostic performance of mRNA quantification has been estimated in a cohort of patients truly representative of those found in the clinical setting. However, to adequately assess the prognostic role, if any, of *AGR2* and *LGR5* mRNA levels in the blood, a larger, more homogeneous cohort of patients is clearly needed. Furthermore, a comparative study with immunofluorescence-based methods such as the Cell Search System is warranted.

## 3. Experimental Section

### 3.1. Patients

Consecutive patients with CRC from the Medical Oncology Unit at the University Hospital in La Coruña (Galicia, Spain) were included in the study. Inclusion criteria were as follows: A confirmed pathological diagnosis of colorectal adenocarcinoma; stage I–III patients with no prior systemic therapy for cancer; or stage IV patients without previous systemic therapy or with confirmed cancer progression after such treatment. Exclusion criteria were defined as follows: Any other previous malignancy; coagulations disorders; platelet count less than 20.0 × 10^9^ L^−1^.

The diagnostic work-up included a clinical examination, blood sampling with CA 19.9 and CEA serum determination, endoscopy (when clinically indicated), thoracic radiograph and computed tomography (CT) scanning of the abdomen and pelvis. Chest CT was performed in patients with rectal tumors and stage IV patients. Patients were followed up with imaging every 8 to 12 weeks to monitor disease progression.

Serum CEA (with an upper limit of normal of 5 ng/mL) and CA 19.9 (with an upper limit of normal of 37 U/mL) levels were determined using enzyme immunoassay (Advia Centaur, Siemens Healthcare Diagnostics) according to the manufacturer’s instructions.

PBs for qRT-PCR analyses were obtained after surgery, before neo-adjuvant chemotherapy or in the presence of active, clinically and radiological advanced progressive disease. At least the first 5 mL of blood obtained was discarded to avoid contamination with epidermal cells.

Controls were consecutively recruited from the patients’ family and relatives. We only excluded controls with a previous history of malignant disease. Thus, controls with different chronic but stable diseases (e.g., hypertension, diabetes mellitus or heart disease) were eligible and consecutively recruited. Controls were selected to include a sex and age distribution that was comparable to the patient group.

This study was approved by the Ethics Committee of Clinical Investigation of Galicia (Spain), and written informed consents were obtained from all patients and controls prior to their inclusion in the study.

### 3.2. Pathological Analysis

Tumors and regional lymph nodes collected during surgery were processed on a routine diagnostic basis. Histological tumor type, depth of invasion and nodal involvement were analyzed, and the disease was staged and graded according to the TNM [41].

Residual disease status at the time of blood sampling was classified as R0 when no residual disease was present after surgery, R1 when microscopic residual disease was found, and R2 in the presence of macroscopic disease. Patients from whom the blood was obtained before the start of neo-adjuvant treatment were categorized as R2.

### 3.3. Processing of Blood Samples and mRNA Isolation

Peripheral venous blood (10 mL) was collected in EDTA-containing tubes. Samples were stabilized within 1 h after withdrawal in guanidinium-based RNA/DNA reagent (Roche, Germany) at 10% (v/v) without cell and plasma separation. An isolation reagent for blood and bone marrow (Roche, Germany) was used for mRNA extraction according to the manufacturer’s protocol with minor modifications [10]. Purified poly(A) + RNA was further processed for qRT-PCR or stored at −80 °C until use.

The RNA concentration was determined based on UV absorption at 260 nm. The A260/A280 ratio was calculated to assess RNA quality and purity.

### 3.4. Reverse Transcription and Quantitative Real-Time PCR

Reverse transcription (RT) was performed on 0.02 μg of mRNA using the Superscript First-Strand Synthesis System (Invitrogen Life Technologies, Carlsbad, CA, USA) as described previously [10]. Real-time PCR analysis was performed using the following primers: *AGR2*-2F, CTGGCCAGAGATACCACAGTC; *AGR2-2R*, AGTTGGTCACCCCAACCTC; *LGR5*-F, CAGCGTCTTCACCTCCTAC; *LGR5*-R, TTTCCCGCAAGACGTAACTC. The *AGR2* and *LGR5* primers amplified 101 bp and 108 bp of the respective cDNAs. Primer pairs were chosen so that the sequences were located in different exons. *Hypoxanthine-guanine phosphoribosyl-transferase 1* (*HPRT1*) was selected as reference gene, as previously reported [8]. *HPRT1* (102 bp) was also used as an internal control to verify the RNA integrity and the efficacy of reverse transcription. Any specimen with inadequate *HPRT1* mRNA was excluded from the study.

The PCR reaction consisted of 10 μL of 2× SYBR Green I Master Mix (Roche, Germany), 1.4 μL of forward (F) and reverse (R) primers at 5 μmol/L (Tib MolBiol, Germany), 4 μL of cDNA and PCR-grade water up to a final volume of 20 μL following the manufacturer’s recommendations. Amplifications were performed in a Light Cycler 480 (Roche Applied Science, Penzberg, Germany).

The maximum number of cycles was 50. If after 40 cycles no fluorescent signal was detected on the amplification plots, the marker mRNA was assumed to be absent from the sample.

We verified that the amplifications and the size of each PCR product were specific by melting curve analysis. Data analysis was performed with Light Cycler 480 Relative Quantification software (Roche Applied Science, Penzberg, Germany). Relative levels of expression were calculated by the 2^−ΔΔCt^ method [42]. Each assay was done at least in triplicate. The average value of the replicates was used as the quantitative value for each sample.

Each assay included marker-positive, marker-negative and no-template controls. RNA analyses were performed with no knowledge about clinical or follow-up data.

### 3.5. Study Design and Statistical Analysis

This project was designed as a prospective early-phase, diagnostic case-control study. The primary aim was to estimate the diagnostic performances of *AGR2* and *LGR5* in blood as clinical biomarkers [43]. Receiver operating characteristic (ROC) curves were constructed by plotting sensitivity (*y*-axis) *versus* 1-specificity (false-positive rate; *x*-axis), and the area under the curve (AUC) was calculated. The optimal cutoff for mRNAs expression level that separates cancer patients and controls was obtained at the point of the maximum Youden index. Binary logistic regression analyses were used to assess for diagnostic suitability of marker combinations.

Secondary aims included the evaluation of *AGR2* and *LGR5* mRNA blood levels in CRC patients according to the disease characteristics and clinical outcomes. Parametric tests were used to analyze the potential correlation between mRNA biomarker expression and clinical and pathological features of study subjects.

PFS was measured as the time between the baseline PB sampling for biomarkers analysis and the documentation of the first tumor progression based on clinical and radiological findings or death of any cause. OS was defined as the time from baseline blood sampling to death of any cause. Patients who were alive and progression-free at the time of analysis were censored by using the time between the baseline PB sampling and their most recent follow-up evaluation. The Kaplan-Meier method was used to estimate PFS and OS. Log-rank tests were used to assess the difference between the survival curves. Hazard ratios (HR) were modeled using Cox proportional hazard regression analysis.

All statistical tests were two-sided, with alpha levels lower than 0.05 considered statistically significant. PASW Statistics 18.0 for Windows (version 18.0; IBM Corporation: Armonk, NY, USA, 2010) was used for statistical analysis.

The study design and results are presented in accordance with the REMARK [44] and MIQE guidelines [45].

## 4. Conclusions

Our findings indicate that the quantitative molecular assessment of *AGR2* and *LGR5* can serve as a surrogate marker of CTC and ISC-like circulating tumor cells in CRC patients. Elevated *AGR2* and *LGR5* mRNA levels in the blood are associated with poor outcome in patients with CRC.

## Figures and Tables

**Table 1:** Patient baseline and clinical characteristics.

	Mean (SD)	Range
**Age (years)**	62.7 (9.6)	31–80
	**N**	**%**
**<60 years**	20	37.0
**≥60 years**	34	63.0
**Gender**		
**Female**	21	38.9
**Male**	33	61.1
**Stage**		
**I–II**	6	11.1
**III**	12	22.2
**IV**	36	66.7
**pT**		
**pT1–pT2**	6	14.1
**pT3**	36	66.7
**pT4**	8	14.8
**pTx**	4	7.4
**pN**		
**pN0**	13	24.1
**pN1**	26	48.1
**pN2**	11	20.4
**pNx**	4	7.4
**M**		
**M0**	18	33.3
**M1**	36	66.7
**Residual disease status**		
**R0–R1**	16	29.6
**R2**	38	70.4
**Number of Metastatic Sites**		
**0**	18	33.3
**1**	25	46.3
**≥2**	11	20.4
**Location of Metastasis**		
**None**	18	33.3
**Liver Only**	23	42.6
**Liver and Other**	11	20.4
**Non-liver Metastasis**	2	3.7
**Grade**		
**Low Grade**	10	18.5
**High Grade**	44	81.5
**Vascular/Perineural Invasion**		
**Unknown**	6	11.1
**No**	24	44.4
**Yes**	24	44.4

**Table 2:** Distribution of clinical and pathological parameters and levels of *AGR2* and *LGR5* mRNA in the blood.

Parameter	*AGR2*			*LGR5*		

	Mean	SEM	*p*	Mean	SEM	*p*
**Age (y)**			0.459			0.128
**<60**	497.0	142.2		22.5	10.0	
**≥60**	367.3	105.1		5.0	5.0	
**Gender**			0.075			0.203
**Male**	291.1	87.9		5.8	3.9	
**Female**	633.7	161.5		22.1	11.7	
**Stage**			0.137*			0.204*
**I–II**	1.1	0.2		0.3	0.05	
**III**	471.3	171.2		0.1	0.08	
**IV**	492.6	113.8		18.4	7.7	
**pT**			0.002^a^			0.915
**pT1–T2**	82.1	57.5		10.7	10.5	
**pT3–T4**	453.7	92.4		12.5	5.8	
**pN**			0.063*			0.309*
**Node Negative**	306.9	162.3		0.26	0.05	
**pN1**	311.7	80.4		13.1	7.01	
**pN2**	795.2	266.3		23.9	16.4	
**M**			0.283			0.024^a^
**M0**	305.4	122.5		0.18	0.06	
**M1**	492.6	113.8		18.4	7.7	
**R Status**			0.671			0.024^a^
**R0–R1**	363.2	156.1		0.13	0.03	
**R2**	442.6	101.7		40.3	7.01	
**Number of Metastatic sites**			0.373*			0.159*
**0**	305.4	122.5		0.18	0.06	
**1**	407.7	145.0		21.5	10.6	
**≥2**	628.4	184.7		12.3	9.3	
**Grade**			0.023^a^			0.024^a^
**Low grade**	183.0	71.9		0.1	0.04	
**High grade**	480.9	102.8		14.6	6.1	
**Vascular/Perineural Invasion**			0.751			0.269
**No**	385.8	100.6		6.6	6.5	
**Yes**	441.2	146.1		18.6	8.5	

*AGR2* and *LGR5*, mean relative expression levels, arbitrary units; SEM: standard error of the median, *t*-test;

* ANOVA;

a *p* values of less than 0.05.

**Table 3:** Progression-free survival and overall survival in relation to clinic and pathological characteristics and blood *AGR2*/*LGR5* mRNA: Multivariate Cox proportional hazard analysis.

		Wald	Hazard Ratio	95% CI		*p*
**Progression free survival**						
Depth of invasion	pT1**–**2/pT3/pT4	1.042	1.430	0.720	2.841	0.307
Lymph Nodes	Negative/Positive	0.834	1.714	0.539	5.445	0.361
Residual disease	R0**–**1/R2	8.047	5.824	1.724	19.68	0.005
*AGR2/LGR5*	Negative/Positive	6.025	2.803	1.231	6.385	0.014
**Overall survival**						
Depth of invasion	pT1**–**2/pT3/pT4	0.741	1.443	0.626	3.322	0.389
Lymph Nodes	Negative/Positive	0.020	1.085	0.348	3.384	0.888
Residual disease	R0**–**1/R2	7.041	7.338	1.683	31.99	0.008
*AGR2/LGR5*	Negative*/*Positive	1.158	1.594	0.682	3.724	0.282

*AGR2/LGR5* negative in blood indicate both mRNA markers below the mean; a positive result indicates *AGR2* and/or *LGR5* above the mean.
